# Jarring Encounters: Discomfort, Disruption, and Dominant Narratives of Suicide

**DOI:** 10.1177/10497323241302653

**Published:** 2024-12-04

**Authors:** Rebecca Helman, Sarah I. Huque, Amy Chandler

**Affiliations:** 1 School of Health in Social Science, 3124 University of Edinburgh, Edinburgh, UK

**Keywords:** suicide, reflexivity, discomfort, feelings, affect, abductive analysis

## Abstract

In researching experiences and understandings of suicide bereavement across diverse communities in Scotland, we expected to hear difficult, distressing, and painful narratives. However, one of the 31 in-depth qualitative interviews that we conducted was particularly and unexpectedly jarring. In this narrative, Freya explained how her ex-partner took his life after she escaped from his domestic abuse. This narrative produces a deep sense of discomfort in the interviewer, as her expectations about suicide bereavement are disrupted. Taking this discomfort as a starting point, we explore what this jarring encounter tells us about dominant and absent narratives of suicide. We interrogate how this narrative of suicide within the context of domestic violence perpetration bumps up against dominant narratives of a “male suicide crisis” and “relationship breakdown,” through which men are positioned solely as “victims.” Drawing on perspectives from feminist, affective, and reflexive qualitative research, critical suicide studies, and an abductive approach to analysis, we explore how attending to uncomfortable feelings that are generated within the research encounter can enable us to develop more complex, nuanced, and messy understandings of suicide.

## Introduction

As Freya^1^ begins to recount her experience of suicide bereavement, I experience a jarring sensation. Her narrative clashes with those of the other bereaved people I have interviewed. Her description of her ex-partner’s suicide as a final act of violence against her after she escaped his domestic abuse, sits uncomfortably alongside narratives of loved ones whose suicides occur within the context of mental illness, economic crisis, and social exclusion. Freya recounts her own sense of discomfort that her experience is not represented in available information about suicide and suicide bereavement. The discomfort produced by Freya’s narrative remains with me, resurfacing every time I return to the transcript, demanding further exploration.

The above is a reflection on doing qualitative research which explores the experiences and understandings of those affected by suicide. The interview encounter with Freya is an example of a “disruptive” narrative, which challenges dominant understandings of suicide, including the kinds of stories we, as researchers, expect to hear in our interviews. In this paper, we use this experience of jarring discomfort as a starting point from which to explore, and query, dominant and absent narratives of suicide. We explore how Freya’s narrative bumps up against dominant notions of a “male suicide crisis” and “relationship breakdown” as contributing to men’s suicide. Drawing on perspectives from feminist, affective, and reflexive qualitative research, critical suicide studies, and abductive analysis, we explore how attending to uncomfortable feelings that are generated within the research encounter can enable us to develop more complex, nuanced, and messy understandings of suicide. Our analysis contributes to a growing body of work on emotional reflexivity as an analytical tool within qualitative health research, drawing on Chadwick’s (2021) “politics of discomfort.”

### Discomforting Feelings in Suicide Research

Researching suicide is emotionally challenging work. It could be considered a “difficult” research topic, with the potential to cause researchers distress (Silverio et al., 2022). Conducting qualitative interviews with people affected by suicide (including those who are bereaved, those who have had suicide attempts, and professionals who support people experiencing suicidality) means hearing stories of distress, pain, shame, and intense loss, among other difficult feelings (Boden, 2017). These narratives reflect that suicide is a major public health challenge, with around 703,000 people dying by suicide each year globally (World Health Organization, 2021), with millions more planning, attempting, or experiencing suicidal thoughts (Centres for Disease Control and Prevention, 2021). These narratives also show how the effects of suicide ripple out across families, neighborhoods, and communities (Cerel et al., 2019).

Perhaps all narratives of suicide can be considered, to some extent, jarring—these are narratives about experiences of “unnatural” or “bad” deaths (Bloch & Parry, 1982). Ongoing shame and stigmatization of suicide make it difficult for people to share these experiences (Owens & Lambert, 2012; Pitman et al., 2018; Sheehan et al., 2018). The desire to end one’s own life can also be seen to jar with normative logics of life as the “normal” or “natural” state of being (Tack, 2019). Therefore, difficult and jarring feelings—including discomfort and dis-ease—can be seen as central to researching suicide. Yet, there is a lack of research which explicitly engages with what suicide research *feels* like or how feelings are implicated in suicide research.

An exception is the work of Boden and colleagues (2016; see also Boden, 2017) who note that the feelings generated within research encounters are epistemically important, as they “provide us with knowledge about the nuanced meanings in our encounters” (Boden et al., 2016, p. 1079). Their work engages with instances of fragility, emptiness, and fear, among other feelings, which emerged during their interviews with people who had attempted suicide, their significant others, and people who have been bereaved by suicide (Boden et al., 2016). Boden (2017) argues that reflexive engagement with researchers’ felt experiences can contribute to the production of more layered, comprehensive, and nuanced accounts of suicide (Boden, 2017). This includes sitting with felt experiences generated during research encounters and *being with* the difficult emotions that participants narrate, when describing their experience of suicide and suicide bereavement (Boden, 2017).

Beyond suicide research, the importance of feelings within the research encounter has also been highlighted by feminist and affect studies research (cf Baaz & Stern, 2009; Baker et al., 2018; Helman, 2023). This work builds on a long history of feminist, Black, and Indigenous scholarship which has advocated for the importance of emotions/affects^2^ and embodied ways of knowing (Ansloos & Peltier, 2022; hooks, 1995; Jaggar, 1989; Lorde, 1997; Million, 2009; Rosaldo, 1984; Stanley & Wise, 1983). Within qualitative research more broadly, the importance of attending to researchers’ emotions throughout the research process has also frequently been highlighted in relation to reflexivity (Finlay, 2002, 2012; Gemignani, 2011; Hubbard et al., 2001; Sharma et al., 2009). Work on reflexivity highlights how emotions are both sources of knowledge and implicated in the production of knowledge (Hedström, 2019). Emotions provide important feedback and insights within the research encounter and can sensitize researchers to key foci and areas in need of further exploration (Eakin & Gladstone, 2020; emerald & Carpenter, 2015; Finlay, 2002; Gemignani, 2011). These forms of reflexivity enable more complex and “messy” explorations that may elucidate and disrupt taken-for-granted and dominant understandings (Kennedy & Gardner, 2022; Sharma et al., 2009). This kind of reflexive engagement can open up explorations of areas that would otherwise remain concealed (Finlay, 2002).

Informed by this work on emotional knowledge and reflexivity, our approach to discomfort draws on the work of Ahmed (2014), who argues that emotions do not exist as individual psychological dispositions but as moving relations between people, objects, bodies, histories, locations, and discourses. Her work calls attention to feelings as “processes of affecting and being affected” (Ahmed, 2014, p. 208). In this paper, we attend to the affect and effects of discomfort, which emerge as Freya narrates her experience of suicide bereavement. This approach is inspired by Chadwick’s (2021) work on the politics of discomfort, which conceptualizes research as affective praxis, in which affects circulate during all phases of the research encounter. These affects:

‘do’ things in research praxis – they mobilise actions, representations, decisions, meanings and interpretations and are thus lively actants in the research production process. Affects ‘turn us on’ or ‘turn us off’ to certain lines of thinking, conceptualising, knowing and making sense. As a result, affects are epistemic resources for knowledge production and theory-making. As researchers, we are ‘affected’ by and affect research participants, fieldwork encounters and data analytic processes. (Chadwick, 2021, p. 557)

In this paper, we engage with discomfort as an unsettling disruption to dominant ways of thinking about suicide and attend to the ways in which this discomfort opens up an alternative narrative about suicide. Drawing on experiences of qualitative interviewing, Sands and Krumer-Nevo (2006) refer to unexpected instances in interviews which produce emotional responses in the researcher as “shocks.” These “shocks” disrupt the implicit expectations of the researcher and represent a clash between different narratives. They note that these “shocks” should be examined in relation to the broader socio-political context in which they are located (Sands & Krumer-Nevo, 2006). Feelings of discomfort, shock, and “being jarred” are an opportunity to reflect on dominant narratives of suicide and the broader context which constitutes these narratives (Zaliwska & Boler, 2019; Zembylas, 2018). Here, we are using the term *dominant narratives* to refer to “pre-existent socio-cultural forms of interpretation” which legitimize existing relations of power (Bamberg, 2005, p. 287). Embedded within particular socio-cultural contexts, these dominant suicide narratives are broadly known and often taken for granted (Abrutyn et al., 2020; Stack & Bowman, 2017). The power and effects of dominant narratives of suicide has been a particular focus within the field of critical suicide studies.

### Critical Suicide Studies: Disrupting Dominant Narratives of Suicide

Our approach draws on work from critical suicide studies, which has been broadly defined as “an open-ended, restless set of ideas, practices, and ongoing struggles which we believe can offer some creative new vantage points with which to ‘think’ suicide into the future” (Costa & White, 2024, p. 2). These ideas, practices, and struggles have emerged in response to the limitations of mainstream approaches to suicidology and suicide prevention, which are dominated by psychological, Western, individualized, and empiricist approaches (Costa & White, 2024; White et al., 2016). Critical suicide studies highlight how suicidology operates as a social practice, cohering around specific assumptions and ideas (Fitzpatrick et al., 2015). A key focus of critical suicide studies has been to question these often taken-for-granted ideas and assumptions about what suicide “is” and how it should be responded to (Costa & White, 2024; Marsh, 2016; White & Morris, 2019).

Within both mainstream suicidology and critical suicide studies, the narratives of those affected by suicide are increasingly recognized as central to developing more nuanced and contextualized understandings of and responses to suicide (Hjelmeland & Knizek, 2010; Marsh et al., 2021; Webb et al., 2024). The narratives of those with direct, or “lived,” experience of suicide and/or suicide bereavement operate as key sites of meaning-making, social identity, and power, yet these narratives may simultaneously reproduce and normalize dominant logics of suicidology (Fitzpatrick, 2016). Dominant narratives frame how suicide is understood, experienced, and responded to, directing and heightening attention to particular elements of suicidal experiences, thus exerting ethical and political power (Fitzpatrick, 2016). Recognizable, coherent, and well-formed narratives about suicide, which circulate in the public domain, offer templates for individuals to describe and explain their own experiences of suicide (Fitzpatrick, 2014). For example, Marsh’s (2010) critique of the “compulsory ontology of pathology” (p. 28) highlights the ubiquitous centering of mental illness in suicide narratives through which suicide is seen to be always related to mental illness. This dominant narrative positions those who die by suicide as “ill,” “irrational,” or “out of their minds.” Concurrently, within many contemporary societies, the narrative of suicide as mental illness can be seen to challenge narratives of suicide as a “selfish” act.^3^ It is, thus, important to ask what kinds of stories can be told about suicide and how personal stories about suicide are shaped by dominant framings, including those produced by practitioners and researchers.

### The “Male Suicide Crisis”

One of the dominant narratives of suicide is that of a “male suicide crisis” (Jordan & Chandler, 2019). This is reflected in the widely repeated assertion that men’s suicides outnumber women’s suicides in almost all countries in the western world (Richardson et al., 2021). The ratio of men’s to women’s suicides^4^ is more marked in high-income contexts compared to low- and middle-income countries, where rates of suicides of men and women are more equal (World Health Organization, 2021). In the United Kingdom, statistics reflect this pattern. In 2022, men’s suicides accounted for three-quarters of the suicide deaths in England and Wales (Office for National Statistics, 2023). In Scotland, the suicide mortality rate was 2.9 times higher for men than women^5^ in 2022 (National Records of Scotland, 2023). In Northern Ireland, men’s suicides represented 76.8% of suicide deaths in 2022 (Northern Ireland Statistics and Research Agency, 2023).

Explanations for why so many men are dying by suicide have focused on cultural norms of masculinity, informed by Connell (2002)’s conceptualization of hegemonic masculinity. This concept, which has been widely debated, proposes that men’s identities are constructed with reference to a dominant, valued idea of what it means to be a man (Connell & Messerschmidt, 2005). A recent qualitative meta-synthesis of two decades of research on men’s suicide found that 96% of the articles related certain cultural norms of masculinity to potential suicide risk (Bennett et al., 2023). Men’s emotional suppression has been repeatedly highlighted as contributing to men’s suicide (Bennett et al., 2023, 2024; Cleary, 2019; de Boise & Hearn, 2017). Men’s suicide is said to be related both to men expressing their emotions less readily and less often than women (e.g., internalizing or repressing emotions) and that when men do express emotions this is more likely to be through “externalizing” in the form of anger, violence, alcohol and drug use, and suicide (Chandler, 2019).

The notion of “relationship breakdown” is also frequently highlighted as contributing to men’s suicide. “Relationship breakdown” was originally conceptualized as being related to women’s suicide or as a “feminized act,” with interpersonal relationships being seen as more important for women (Canetto, 1993). However, more recent research has highlighted that the breakdown of interpersonal relationships (including through separation, divorce, and separation from children) may be more likely to contribute to men’s suicide (Bennett et al., 2023; Owens et al., 2008; Scourfield, 2005; Scourfield & Evans, 2015; Wyllie et al., 2012). Explanations for why relationship breakdowns are more likely to cause distress for men include the idea that men receive more care and derive more benefits from (heterosexual) relationships; that men may have fewer forms of emotional support outside of their intimate relationships; and that men may be more likely to be separated from their children following divorce (Scourfield & Evans, 2015). While the notion of “relationship breakdown” may point to an important source of men’s distress, this broad category tells us very little about the nature and circumstances of men’s intimate relationships and the conditions under which they might “breakdown.”

Notions of a “male suicide crisis”—including men’s emotional suppression and relationship breakdown—are often mobilized in public and academic discourse to position men as “victims,” “disempowered,” or “wounded” (Fitzpatrick et al., 2022; Jordan & Chandler, 2019; Scourfield, 2005). In some instances, these narratives are deployed to construct men as “disenfranchised” within more gender equitable environments (Jordan & Chandler, 2019). While not dismissing men’s distress, critical scholars have argued that there is a need to engage in more complex ways with notions of a “male suicide crisis,” including attending to how men are positioned in different ways to power and social inequality (Chandler, 2019; Fitzpatrick et al., 2022; River & Flood, 2021; Scourfield, 2005). This includes attending to men’s intersecting identities, for example, as queer, minoritized, or economically deprived (Chandler, 2019). In her work with working-class white men in Scotland, Chandler (2019) has argued that the notion of thwarted privilege, where men are unable to attain the expected rewards of masculinity, can help us to understand some men’s experiences of suicide. For some men, who have been “stopped” or derailed from the lives they expected, suicide attempts are a way for them to still “do.” Some of these attempts occur within contexts of domestic violence and therefore are also entangled with control and violence against others (Chandler, 2019).

Currently, there is very limited research that has engaged with men’s suicide within the context of domestic or family violence perpetration (Bennett et al., 2024; Chandler, 2019; Fitzpatrick et al., 2022). Perhaps this is because these kinds of explorations jar with dominant explanations of men as “victims” within the context of a “male suicide crisis.” However, the limited research which does exist highlights that in some instances men’s suicides or attempted suicides occur within the contexts of violence against others (Chandler, 2019; Fincham et al., 2011; Fitzpatrick et al., 2022; River & Flood, 2021). For example, Scourfield and colleagues (2012), in an analysis of 100 coroner reports of suicide in England, found that 23 cases (22 of which were men) involved some kind of abusive behavior by the deceased. In the United States, a quantitative study of suicides in North Carolina found that intimate partner violence contributed to 4.5% of suicides—of these, 73% were men who had perpetrated intimate partner violence prior to their suicides (Kafka et al., 2022). In Australia, Fitzpatrick and colleagues (2022), also analyzing coronial reports, found that threats of suicide were reportedly used as a form of coercive control by men within the contexts of divorce and custody battles, for example, by men threatening suicide if their partners ended the relationship. Their qualitative analysis of medico-legal reports demonstrates that in some instances suicide was directed toward punishing and exacting revenge, blame, and guilt on female partners and that some men left spiteful notes. A recent analysis of domestic abuse perpetrators in England and Wales (92% of which were men) found that the rate of suicide among this group was 23 times greater than in the general population (Knipe et al., 2024). This research suggests the need to further explore how both men’s power and powerlessness may be implicated in suicide and to move beyond ideas of men as merely “victims” (Fitzpatrick et al., 2022).

Following this developing evidence on the role of domestic violence in men’s suicide, we analyze Freya’s narrative about her ex-partner, who died by suicide following her leaving their relationship as a result of his abuse. We explore how discomfort circulated within the interview encounter, dis/orientating us toward Freya’s narrative as an interruption of dominant narratives of “relationship breakdown” and “male suicide crisis.”

## Methods

Freya’s interview was one of 61 interviews with people affected by suicide conducted as part of the *Suicide Cultures: Reimagining Suicide Research* project. Suicide Cultures sought to explore the social contexts and cultural meanings of suicide across three diverse areas of Scotland.^6^ The project aimed to extend understandings of suicide beyond psychological and psychiatric perspectives and to situate suicide as a social and cultural phenomenon, shaped by the socioeconomic inequalities, gender relations, and social identities which exist within specific communities.

Interview participants included 19 people who had attempted suicide, 31 people bereaved by suicide, and 33 people working in professional capacities to support people experiencing suicidality and suicide bereavement.^7^ This paper focuses on the narratives of those bereaved by suicide, including parents, friends, siblings, children, aunts, partners, and ex-partners. The majority of people bereaved by suicide identified as White Scottish, female, and heterosexual.

We used an open-ended interview approach, guided by broad questions, to allow participants to highlight the elements they felt were most salient to their experiences. In interviews with people bereaved by suicide, we asked people to tell us about the person who had died, their relationship with them, their death, the impact their death has had, the support they have received in the wake of their bereavement, and what they think can be done to prevent suicide. Interviews lasted between 30 minutes and 2 hours. Researchers met with participants for a pre-interview, to discuss what the interview would entail, go through the consent form, and give participants an opportunity to ask any questions. Interviews were scheduled for a time and place that was convenient and comfortable for participants. All participants gave written consent^8^ prior to their interview. Some interviews were conducted online, but the majority were conducted in person. Participants were given a £20 voucher for participating. Prior to the interview, researchers discussed a care plan with participants, in the event that they found the interview distressing. Alongside this, participants were also provided with a list of relevant organizations where they could seek support.^9^ Ethical approval was granted by the University of Edinburgh’s School of Health in Social Science Ethics Committee.

Given the sensitive and potentially distressing nature of the research, establishing procedures for researcher well-being was of paramount importance. This included limiting the number of interviews per day and debriefing with other team members following interviews. Despite all these procedures, the effect of conducting the interviews was still deeply distressing at times, and we often found ourselves needing to take time away to recover, both after some interviews and while analyzing the transcripts.

Our analytical approach was informed by Timmermans and Tavory’s (2022) abductive analysis. This approach argues for the importance of “surprises” within data analysis and urges researchers to explore what these surprises mean and the consequences that they produce (Timmermans & Tavory, 2022). They note that “outliers” are important because they require a rethinking of why these examples do not “fit” and offer opportunities to defamiliarize the familiar (Timmermans & Tavory, 2022). Similarly, Chadwick (2021) advocates for engaging with the discomfort produced by “outliers” or “problematic” accounts which do not fit into homogenous, coherent categorizations. Rather than dismissing Freya’s account as a mere “outlier,” we explore the discomfort generated by Freya’s account as a dis/orientation which enables us to explore both dominant and absent narratives of suicide.

All interviews were initially coded using an open coding approach. A broad list of thematic codes was developed by the research team through ongoing engagement with the data. This coding procedure allowed us to examine the dominant explanations of suicide that were presented in participants’ narratives and to confirm the initial feeling that Freya’s narrative jars with these explanations. A secondary round of analysis was conducted with the relevant thematic codes, focusing specifically on interpersonal relationships, violence, and notions of people who die by suicide as “victims.” Through this analytical process, one other example where the person who died by suicide had perpetrated domestic violence against their partner, and where their relationship with the partner was narrated as contributing to their suicide, was identified. This example is explored below, alongside an in-depth engagement with Freya’s account. We also present extracts from other bereaved participants to highlight dominant explanations of suicide and the ways in which Freya’s narrative jars with these explanations. Alongside extracts from interview transcripts, our analysis is also informed by extracts from the first author’s (RH) research diary, in which she recorded reflections both directly following the interview and during the analysis process.

Our analysis attends to what researching suicide feels like and what these feelings enable us to understand about suicide—taking up Boden and colleagues’ call for a reflexivity of feelings in suicide research (Boden, 2017; Boden et al., 2016). We explore how RH^10^ is affected by Freya’s narrative and how these affects move our analysis in particular ways (Finlay, 2002; Hubbard et al., 2001). This involves tracing how we *feel* our way through our research on suicide and how we are affected by researching suicide (Allan & Arber, 2018; Laliberté & Schurr, 2016). Our approach also explores the multiple layers of feeling that emerge while researching suicide and what they tell us about suicide (Boden, 2017). In our engagement with discomforting affects and effects of Freya’s narrative, we explore how we may be primed to hear particular dominant narratives about suicide (Salway & Gesink, 2018).

## Attending to Discomforting Narratives of Suicide

Freya is in her sixties and lives in a large urban area. The interview with Freya took place in our project office on a particularly rainy day and lasted 72 minutes. When I asked Freya to elaborate on her experience of suicide bereavement, she explained:

I was in an abusive relationship for […] about ten years I guess, and my daughter […] we managed to escape about three months before that. And so, the suicide was, sort of, the culmination of the aftermath of that. And there had been police involvement and there had been an arrest and a release on bail and then the suicide. So, for me it was actually a relief. I mean it was a shock… but it was a relief. It was horrendous for my daughter […] she’s still struggling with it to a great extent. She’s recovered a lot but I can see it’s still affecting her.

As Freya recounts her experience of bereavement, I feel an uncomfortable weight in my stomach—this is not the sense of dread or grief I have experienced in other interviews with people bereaved by suicide. Instead, it is an uncomfortable sense that I had not anticipated the kind of narrative that Freya shares with me. This discomfort highlights the edge of my understanding of suicide within the context of domestic violence (Boden, 2017). Freya’s narrative of suicide bereavement within the context of leaving an abusive relationship jars with dominant narratives of “relationship breakdown” and a “male suicide crisis,” through which men are positioned as “victims.” While her sense of shock is echoed by many of the other bereaved people we interviewed, her sense of relief bumps uncomfortably against the emotions other bereaved participants describe—including profound grief, emptiness, guilt, and shame.

Later in the interview, it became clear that my sense of discomfort was (at least partly) related to Freya’s own discomfort in trying to relate her experience to “normative” experiences of suicide. This is an example of how my sense of discomfort allows me to interpret Freya’s experience—and how the interaction between Freya’s and my discomfort helps to make visible the edges of “normative” experiences of suicide. Freya described her own sense of discomfort in the wake of her bereavement when she was searching for more information to try and understand what had happened:

But at the time all I wanted to do was find out more about suicide, why do people do these things, because I was so angry as well. And there was a lot about, you know, people in despair and about young people but nothing about people who did it as a, kind of, manipulative tactic, or anything like that.

The idea of suicide as a tragic act of despair, which Freya highlights here, looms large in dominant and public understandings of suicide. Many of the bereaved people we interviewed articulated these kinds of narratives, through which the person who has died by suicide is presented as a “victim” overcome with despair. This narrative of tragic despair has been bolstered by dominant constructions of suicide as related to mental illness, and mental illness as always a tragic cause of suicide. For example, Susan described struggling with her son Luke’s erratic, disruptive behavior and substance use issues, which also negatively affected her other children. Yet, in explaining Luke’s suicide, which occurred when he was in his twenties, she constructed him ultimately as a “victim”:

And, I don’t know, he had a … I mean, his life was rubbish really. It was a struggle. And he wasn’t looked after by the NHS. And he wasn’t looked after, I mean, he was looked after by us but at points he wasn’t because we couldn’t look after him, you know. Literally, like, he’d have to get put out the house. And he always found his way to somewhere, to a hostel or something and then came back. And latterly he had his flat, but it was kind of sad, you know. It was, it was always sad, we always felt sad when we saw him.

Here, Luke is constructed as being let down and not cared for adequately by both the NHS and his family. The feelings of sadness that Susan mentions in relation to her son also serve to position Luke as an object of pity. This construction of the person who died by suicide as a victim was a dominant narrative among the bereaved people we interviewed, with the majority articulating this kind of narrative in relation to the person they had lost.

Freya’s narrative jars with this “victim” narrative. In contrast, Freya narrates her ex-partner as a perpetrator of violence, his suicide entangled with other forms of violence that he subjected her (and her children) to:

I guess he was what you would probably call a typical controlling, manipulative type of person who basically took control of everything and didn’t allow me or my daughter to have a life […] It was horrendous for us […] There were … he always made threats about what he would do to us if you know, if I tried to leave […] So, there was always that threat of violence in some way. And when we did leave, he tried to come after us and my daughter and myself were in a women’s refuge for several months […] But fortunately the police […] they arrested him […] Yeah, so I [sighs] … my feeling is that because he couldn’t find us and couldn’t harm us, the only option he had was to turn his rage on himself […].

As Freya recounts this part of the story, I feel myself becoming angry. Freya’s experiences of abuse connect affectively with my previous experiences of researching sexual violence and of hearing endless stories of women subjected to violence by men. In this context, I find it hard to empathize with Freya’s partner, in the way I have empathized with the other people who have died by suicide whose stories I have heard. In part, this is related to Freya’s refusal to narrate her ex-partner in a sympathetic way, given the violence he has subjected her to. But my inability to empathize with Freya’s ex-partner also feels uncomfortable. I feel I should be able to empathize with those who die by suicide—in order to be a “good” suicide researcher.

At this point in the interview, I glance down at my interview guide and see the term “loved one” repeated in many of the questions (“Can you tell me about your loved one?”, “Can you tell me about your loved one’s death?”, “Can you tell me how your loved one’s death has affected you?”). The term “loved one” feels uncomfortably inappropriate within the context of Freya’s narrative and indicates a particular assumption made by us as the research team about the kinds of relationships that our participants have with the people who have died by suicide. As MacArtney (2024) argues, the use of the term “loved one” is loaded with expectations that may delegitimize more complex relationships between a bereaved person and someone who has died. Freya’s framing of her experience of suicide within the context of domestic abuse, and the impact this abuse has had on her, is a disruption of the simplistic use of the term “loved one” and points to the complexity of relationships that may exist before and after suicide. Freya articulated how the complicated relationship that her daughter had with her father shaped her experience of processing his death in counselling:

There wasn’t any way for her to express her anger or … yeah, there was just something … it would have been fine for someone who had a great relationship with their father but for someone who had a very ambivalent relationship with him it was difficult.

Here, Freya’s narrative points to the potential consequences of assuming a particular and specifically positive relationship between the person who has died and those left behind within the context of suicide postvention. These assumptions may prohibit the articulation of more complex relationships and feelings. While feelings of anger are frequently articulated by those bereaved by suicide (Saha et al., 2017; Shields et al., 2017; Spillane et al., 2018), the complexity of bereavement within the context of conflict and violence is largely absent. While Freya’s experience could be broadly captured under the explanation of “relationship breakdown” within the context of a “male suicide crisis,” this description does not attend to the abusive dynamics of the relationship, the harm that Freya experienced within the relationship, and how these elements shape her experience of suicide bereavement.

In her interview, Freya also highlighted how her ex-partner’s identity was transformed through his death by suicide:

Oh it was all … he suddenly became a saint […] [sighs] his close friends had always had this kind of ambivalent relationship with him because they knew he could be a total prick and … but they also … you know, he was very sort of […] as long as he was getting all the positive attention everything was fine. So, there was that side of him too and people were very drawn to that. So, yes, we were demonised for a while and he became a saint […] And, yeah, his brother in particular became quite negative towards us, yeah. So, it was difficult.

Again, this highlights the dominance of “victim” narratives of suicide and how those who die by suicide may come to be positioned in more positive ways due to the circumstances of their deaths. Simultaneously, Freya highlights how she is “demonized” in the aftermath of his death. Here, Freya implies that she is seen to be to blame for her ex-partner’s suicide. This construction of (predominantly) female partners as contributing to men’s suicide is one of the consequences of narratives of “male suicide crisis” and “relationship breakdown.” This narrative was also present in the interview with Brody, a man in his forties who lives in a remote small town. He described how he originally blamed his brother’s ex-partner for his brother, Evan’s, suicide.

When it first happened, in my head I blame, I did … not just her, but his ex-partner, I thought it was her. I said, it’s because of the kids, it’s not … but the more I went into it, it’s because he was abused, and it’s … Aye, so I thought … I sort of … blamed her […] I think it was … that was my first thought, because they were, basically they were at each other’s throat all the time, sort of thing, it was all over Facebook and stuff like that. He was getting the kids one minute, he wasnae getting the kids the next, so she had appeared at the house on the day, and that’s how [other brother] first went looking for [Evan], because they had fell out […] so she had said, you’d better check on Evan […] so my thinking is, after they fell out, that’s when he’s done it.

However, Brody described how it became clear that there were other factors that he later felt contributed to Evan’s suicide:

so that was my initial thought, but as I said, once I went through things and that, it’s the abuse. […] He was basically … a lot like me, obviously brought up together, he fell into drink and drugs and he had a lot of problems with an ex-partner, so had kids together and had a lot of problems. That was his life just before he died, but going back to his childhood, he was abused by [family member], something I didn’t find out ‘til a lot of years later.

Here, Brody describes how, while he initially thought Evan’s problems with his ex-partner were the “cause” of his suicide, it later became clear that Evan was struggling with other issues—including a history of childhood sexual abuse and the death of his mother 2 years previously. This is an example of how “relationship breakdown” operates as a dominant “common-sense” understanding or script for men’s suicide (Fincham et al., 2011), at times obscuring further complexity.

As mentioned above, through coding all interview transcripts, we became aware of another narrative that had some clear overlaps with Freya’s. This was the narrative of the suicide of Joshua, who died in his thirties, told to me by his family member Abigail, who is in her sixties and lives in a large urban area. Abigail’s narrative also described Joshua’s perpetration of violence against his wife, culminating in him being arrested and jailed. Following his release from prison, he had reconnected with his wife and was attempting to re-establish their relationship. Abigail described Joshua’s suicide as occurring within the context of being unable to re-establish this relationship. While bearing many similarities to Freya’s experience, we did not originally make the connection between these narratives. Upon further reflection, it appears this was because of the differences in the ways these two men were constructed in the interviews. As has been highlighted above, while Freya’s ex-partner is constructed as a (unsympathetic) perpetrator of violence, Abigail’s construction of Joshua was much closer to the construction of a passive victim who was not responsible for all the terrible things that happened to him (or that he did to other people), similar to Susan’s construction of her son Luke. Abigail framed Joshua’s life as a series of tragic events, beginning in his childhood, frequently emphasizing his position as a “victim.” For example, Abigail narrated Joshua’s domestic violence as being related to his use of drugs.

And then he started going out with people and mixing with the wrong people, and taking drugs and things like that. And then he would come back and then he would argue with his wife […] things really got bad. And he must have used too many drugs or been on them for so long […] And when [his wife] got back home, he’d taken so many drugs, he started beating her. And he beat her so violently […] I’ve never seen anybody … I’ve never seen anything like that, I hope I never see it again.

Here, the narration of Joshua’s drug use serves to diminish his responsibility for assaulting his wife. Joshua’s responsibility for his drug use is also diminished by Abigail attributing this to him “mixing with the wrong people.” Similarly, in her narrating of Joshua’s suicide, Abigail constructs Joshua as a “victim” in relation to his relationship with his wife. Abigail explained that Joshua’s suicide occurred as a result of him not being able to re-establish his relationship with his wife. She explained that Joshua’s wife had been in a relationship with someone else during this time and insinuated that Joshua’s wife’s desire to re-establish the relationship was not genuine. Abigail describes what he did on the day of his suicide:

This letter that he’d written […] he went to […] his wife’s house […] he was ringing the bell, but nobody opened the door. They were told not to open the door. And he folded this letter up and put it through the letterbox […] And he went away and sat in his car […] And he sat in that car and maybe he waited, we don’t know […] he just stayed in his car, and he plugged whatever it is that they do, so that the fumes come in.

Abigail described how the place where Joshua died was a significant place for him and his wife. She also explained that the letter that Joshua had written to his wife was torn up by his wife’s father but that when the police returned it some (small) parts of it could be read:

And I could see, the shreds were still there […] But the wee bits that they had managed, it was all … “I don’t want to go, but if you come to me and”…

This note bears similarities to some of the messages sent by men in Fitzpatrick and colleagues’ (2022) study in which they threaten suicide to prevent their partners from leaving them. The recovered parts of the note, combined with Abigail’s narrative about the context of Joshua’s suicide, present the ongoing relationship issues with his partner as a key “cause” of his suicide. This is similar to Freya and Brody’s narratives, where female partners are positioned as contributing to or “causing” men’s suicide. It is important to note that Abigail strongly condemned Joshua’s violence against his wife, as well as patriarchal violence and control more broadly. We can understand Abigail’s portrayal of Joshua as a “victim,” despite his violence, within the context of suicide as a shocking, jarring, “bad” death which transforms those who die into “victims,” as Freya demonstrated in relation to the reactions of the friends and family of her ex-partner following his death.

## Discussion

Our analysis has highlighted how the discomfort produced by Freya’s narrative troubles dominant understandings of men’s suicide in relation to notions of a “male suicide crisis” and “relationship breakdown.” Sands and Krumer-Nevo (2006) note that the expectations of dominant narratives can prevent researchers from hearing more unfamiliar, complex, and “messy” narratives. Rather than positioning Freya’s narrative as a mere “outlier,” we have engaged with how the shock produced by hearing her narrative has allowed us to explore how dominant narratives frame suicide in particular ways, while excluding and silencing other understandings (Fitzpatrick, 2016; Marsh et al., 2021). While Freya’s narrative was initially experienced as a jarring “outlier,” our analysis has revealed that her narrative overlaps in significant ways with the narratives drawn on by other participants. In particular, the similarities between the suicides of Freya’s ex-partner and Joshua have allowed us to explore how people who die by suicide are likely to be positioned as “victims” and how these kinds of narratives may conceal more complex and even abusive elements of men’s suicide.

In line with previous work on emotional reflexivity and the importance of engaging with feelings within the context of suicide research, attending to the discomfort produced within the interview encounter allows us to get closer to Freya’s own experience of discomfort in relation to her suicide bereavement (Boden, 2017; Boden et al., 2016). Attending to how we are moved within the context of suicide research has allowed us to move beyond dominant and common-sense understandings of suicide.

While engagement with the discomfort that permeated the interview encounter with Freya has been deeply productive for exploring dominant and absent narratives of suicide, our analysis has generated new kinds of discomfort. Ian Marsh and colleagues (2022) have noted that representing perspectives which challenge existing norms of suicide research and prevention is uncomfortable work. We are deeply cognizant of the dominant narratives which construct people who attempt or die by suicide in stigmatizing, unempathetic, and problematic ways, including narratives of suicide as “selfish” or “attention seeking.” In attempting to trouble dominant narratives of suicide, our intention is not to demonize men, including those who perpetrate acts of violence against others, or to reproduce notions of suicide as “selfish” and “attention seeking.” We do not wish to portray these men in unempathetic ways but rather to reflect on how framing them *only* as “victims” presents a partial understanding of their suicides and the effects their deaths have on those who are left behind.

We also recognize that making sense of suicide is a deeply painful task for those who are bereaved and that the meanings that those who are bereaved by suicide make are embedded within the context of intimate relationships with those who have died. It is, thus, unsurprising that family members may portray those who have died in more sympathetic ways than an ex-partner who was abused. In their work with people bereaved by suicide, Owens and colleagues (2008; Owens & Lambert, 2012) noted that constructing the identities of those who have died by suicide is a form or moral work or repair, in the wake of an “unnatural,” “bad,” or “shameful” death. This moral work is both about the identities of those who have died and the degree to which they were “responsible” for their own deaths, and the identities of those who are bereaved and how they are implicated in the suicide of their family member/friend/(ex)partner. The discomfort around how to portray those who have died by suicide also points to the (im)possibilities of speaking ill of the dead. As we have shown in our analysis, the narrative of people who die by suicide as “victims” is a powerfully dominant one, which may constrain more complex and “messy” representations of those who die by suicide, including within contexts of relationship difficulties and violence.

The discomfort that has been generated through working with Freya’s narrative also sheds important light on the “feeling rules” (Hochschild, 1979) of suicide research and qualitative health research more broadly (see also Dickson-Swift et al., 2009). The discomfort produced by the “failure” of the first author to empathize with Freya’s ex-partner in the same way that she empathized with other people who had died by suicide is produced within a context in which we, as “good researchers,” are expected to empathize with those who die by suicide. This is another effect (and affect) of dominant narratives of suicide which position those who die by suicide as “tragic victims.” The broader social context of shame and stigma related to suicide may intensify the desire to empathize with those who die by suicide and portray them in more “flattering” ways. This is not to suggest that those who die by suicide are not deserving of empathy or that categories of “victims” and “perpetrators” are fixed or mutually exclusive but rather to highlight how this empathy imperative shapes the ways in which researchers engage with and represent those who died by suicide. In a similar way to people who are bereaved by suicide avoiding speaking ill of those who have died, we as researchers may resist “writing ill of the dead” within the context of suicide. The “emotion rules” of suicide research are inextricably linked, then, to the production of knowledge about suicide.

Engaging with and probing feelings of discomfort within the research process may offer opportunities to explore and trouble our own assumptions and enable us, as researchers, to produce more uncomfortably nuanced narratives of suicide, which move beyond simplistic portrayals of those who die by suicide as always and only “victims.” This is particularly important within the context of the “male suicide crisis” and emerging evidence that some men’s suicides occur within the contexts of domestic violence and may be used as a tool to exert control over and inflict pain on (often female) partners (Fitzpatrick et al., 2022; River & Flood, 2021; Scourfield et al., 2012), as Freya’s narrative also powerfully illustrates. Our analysis builds on emerging work on the role of domestic violence in men’s suicide, which has predominantly focused on the review of death records (Chandler, 2019; Fitzpatrick et al., 2022; Knipe et al., 2024; River & Flood, 2021; Scourfield et al., 2012), through a focus on the experiences of those bereaved by suicide. While perhaps uncomfortable to acknowledge, our analysis shows that in some instances suicide may be tied up with men’s practices of power within certain spaces and relationships rather than—or perhaps as well as—being evidence of men’s powerlessness (Fitzpatrick et al., 2022).

Thus, there is a need to move beyond constructing men’s suicide as a homogenous category and to engage in more intersectional ways with how particular men are positioned in relation to suicide, violence, and relationship breakdown (Chandler, 2019). These more complex engagements with suicide have the potential to inform more relevant and appropriate suicide prevention efforts, both in relation to those who attempt suicide and in supporting those who are bereaved by suicide within the context of domestic violence. Our analysis shows that the intersection between domestic violence and men’s suicide demands further attention in both suicide research and prevention. More broadly, exploring jarring narratives offers important opportunities to unsettle taken-for-granted and common-sense understandings of suicide. Within the Suicide Cultures project, we will continue to attend to these unsettling encounters.

As we have attempted to highlight, this kind of emotional reflexivity is challenging. We echo other researchers who have noted that this work can be a deeply uncomfortable, laborious, and even painful task (Finlay, 2002; Gemignani, 2011; Helman, 2023; Kinitz, 2022). This work requires us to vulnerably expose our own feelings and assumptions and to “[stay] with the trouble” of these feelings and assumptions (Haraway, 2016, p. 1). This adds an additional layer of emotional labor, within the context of the already demanding nature of suicide research, and thus requires sufficient emotional support from research institutions (Kinitz, 2022; Sampson et al., 2008). This emotional support may include debriefing within the research team, individual debriefing, time away from the data, and the difficult feelings it can generate, as well as funding to support therapeutic and wellness interventions for researchers which are built into research proposals.

## Conclusion

Informed by work on emotional reflexivity and specifically the role of “shocking,” surprising, or uncomfortable feelings within the research process, we have demonstrated how attending to these unsettling moments can provide important insights within the context of qualitative health research. Our approach has demonstrated how attending to jarring encounters enables us to trouble dominant narratives of suicide. We have shown how challenging these narratives is discomforting work and requires reflection on how our own assumptions throughout the research process are informed by dominant and common-sense understandings of suicide. Through an engagement with the discomfort that is produced in the encounter with Freya, we have sought to unsettle these understandings of “male suicide crisis” and “relationship breakdown” in order to demonstrate how these narratives close down more complex, messy, and nuanced ways of understanding, engaging with—and responding to—suicide.

## Notes

1. Pseudonyms have been used for all participants to protect their identities. Some personal details and details from participants’ narratives have also been changed or omitted to make participants less identifiable.

2. There has been considerable debate about the distinctions between “emotions” and “affects,” with some scholars proposing that affects refer to bodily intensities, while emotions refer to social categories of feeling (Greyser, 2012). However, other scholars have highlighted the ambiguities of these categories and difficulty of maintaining a separation between them (Cvetkovich, 2012; Koivunen, 2010). We refer to emotions, affects, or feelings to mean sensations that resonate in the body and are entangled with the relational and socio-political context in which they arise.

3. We acknowledge that narratives of suicide as “selfish” continue to circulate and construct those who die by suicide and those bereaved by suicide in problematic ways.

4. Most suicide statistics draw primarily on official data sources which use binary sex categories of “male” and “female.”

5. This report indicated a 10% increase in women’s suicides for 2022, with a 2% decrease in men’s suicides (National Records of Scotland, 2023).

6. The three broad areas of the project have been pseudonymized to protect people’s identities.

7. Many of the people we interviewed belonged to more than one of these categories.

8. This included consent for their accounts to be used in academic publications and other research outputs.

9. As we explore below, standardized forms of support (e.g., helplines) may not always be helpful for participants, enhancing the need for more personalized approaches to care within the research encounter.

10. The first author, Rebecca Helman, conducted the interview and led on the analysis for this paper.
